# Architects and Bath Rooms

**Published:** 1878-04

**Authors:** 


### ARCHITECTS AND BATH ROOMS.

WE do not believe there is one Architect in the United States known to favorable fame who is capable of doing either one of two things, viz.:

Of erecting a public building that will not leak, or,

Of constructing a dwelling heated by a furnace which cannot take fire.

Perhaps they are not to blame—the odium may be due to parsimonious landlords. But wherever the fault is chargeable, intelligent and observing men know very well that there is scarcely a public building in New-York of late erection which has not leaked, or which does not now leak. And who knows a perfectly safe flue in all New York? Is there an Architect who understands the philosophy of a Bath-Room? The room of all others, except a permanent sitting room, which most needs a flue or register, or other heating apparatus attached to it, is the "Bath Room," and yet we have never seen a bath room with such an attachment.

Let us for a moment lay aside all book and newspaper knowledge, all preconceived notions, and consult our feelings in the operation of that kind of bathing whose object is to make the body clean of the grease, scales, dust, &c., which are constantly accumulating on its surface.

We all know that cold water will not make the hands clean, nor will hard water, even if it is warm. Hence when we wish to wash ourselves very clean, we use warm water with soap, and if we can get it, rain or cistern, or snow water.

With the present habits of civilized life, comparatively few persons among the middle and upper classes of society have vitality enough to make a cold bath advisable: they have not that "reaction" which gives to a cold bath its highest advantage, hence even the most rabid cold-waterist does not advise cold baths under such circumstances. Then when we take into account how many children there are who are too young for a cold bath, that old have not the stamina for it, whilst to that large number, neither infants, nor aged nor young people, but "children" who roll about in the dust, and mud, and snow, who sprawl upon the floor and dabble in water and dirt, the bathing which is most needed, is a cleansing operation; nothing short of soap, warm water, and a bristle brush will meet their demands, so that after all, especially in winter time, by nine persons out of ten in the whole community of those who practise bathing, the tepid or warm bath is what is needed. In fact, only the very small class of persons who are robust "can stand" a cold bath in winter, and in our opinion such persons do not need it. *If you are well, let yourself alone as to remedial means, for you can't be better than well.* Personally, this is our theory and practice too, we never had a cold bath but once, since boyhood, and that made us sick, and we shudder at the thought of a cold bath ever since.

